# Improved production of germacrene A, a direct precursor of ß-elemene, in engineered *Saccharomyces cerevisiae* by expressing a cyanobacterial germacrene A synthase

**DOI:** 10.1186/s12934-020-01500-3

**Published:** 2021-01-07

**Authors:** Weixin Zhang, Junqi Guo, Zheng Wang, Yanwei Li, Xiangfeng Meng, Yu Shen, Weifeng Liu

**Affiliations:** 1 State Key Laboratory of Microbial Technology, Shandong University, No. 72 Binhai Road, Qingdao, 266237 People’s Republic of China; 2 Environment Research Institute, Shandong University, Qingdao, 266237 People’s Republic of China

**Keywords:** ß-elemene, Germacrene A, Germacrene A synthase, Site-directed mutagenesis, Metabolic engineering

## Abstract

**Background:**

The sesquiterpene germacrene A is a direct precursor of ß-elemene that is a major component of the Chinese medicinal herb *Curcuma wenyujin* with prominent antitumor activity. The microbial platform for germacrene A production was previously established in *Saccharomyces cerevisiae* using the germacrene A synthase (LTC2) of *Lactuca sativa*.

**Results:**

We evaluated the performance of LTC2 (*Ls*GAS) as well as nine other identified or putative germacrene A synthases from different sources for the production of germacrene A. *Av*GAS, a synthase of *Anabaena variabilis*, was found to be the most efficient in germacrene A production in yeast. *Av*GAS expression alone in *S. cerevisiae* CEN.PK2-1D already resulted in a substantial production of germacrene A while LTC2 expression did not. Further metabolic engineering the yeast using known strategies including overexpression of tHMGR1 and repression of squalene synthesis pathway led to an 11-fold increase in germacrene A production. Site-directed mutagenesis of *Av*GAS revealed that while changes of several residues located within the active site cavity severely compromised germacrene A production, substitution of Phe23 located on the lateral surface with tryptophan or valine led to a 35.2% and 21.8% increase in germacrene A production, respectively. Finally, the highest production titer of germacrene A reached 309.8 mg/L in shake-flask batch culture.

**Conclusions:**

Our study highlights the potential of applying bacterial sesquiterpene synthases with improved performance by mutagenesis engineering in producing germacrene A.

## Introduction

Terpenoids (terpenes/isoprenoids) are the most abundant and the largest class of natural products that are widely used as pharmaceuticals, herbicides, flavorings, fragrances, and biofuels [1, 2]. Natural extraction of trace and valuable terpenoids from their native sources, mostly plants, requires large amounts of plant materials, tedious procedures and high production cost, which is far from satisfying their market demand. With development of metabolic engineering and synthetic biology, platform microorganisms have been employed as cell factories for efficient synthesis of terpenoids. The budding yeast *Saccharomyces cerevisiae* is one particularly promising host for terpenoid production due to its generally recognized status as safe and robustness in fermentation. Specifically, *S. cerevisiae* has a native mevalonate (MVA) pathway that can be exploited to effectively supply isoprenoid precursors.

Several metabolic engineering strategies have been well established to increase the yield of terpenoid products in *S. cerevisiae* [3–6]. One critical step is to increase the precursor supply, which can be achieved by overexpressing a truncated version of the rate-limiting HMG-CoA reductase 1 (tHMGR1) [7], the farnesyl pyrophosphate (FPP) synthase ERG20 [8], and the transcription factor UPC2-1 [9, 10]. Together these strategies promote flux through the MVA pathway in cases of synthesizing all classes of terpenes. Moreover, downregulation of the competing squalene synthesis pathway by replacing the native *ERG9* promoter with repressive promoters like *MET3* [8] or *HXT1* [11], is another effective strategy to increase the supply of FPP in mono-, di- and sesquiterpenes production. In addition to boosting precursor supply, highly efficient terpene synthases represent a crucial “pulling” step for biosynthesis of target products with high yield. To this end, strategies such as screening efficient synthases from various sources, enhancing enzyme expression via codon optimization, improving enzyme properties via protein engineering, and expressing fused enzymes catalyzing adjacent reactions, have been employed.

Terpenoid synthases are responsible for the cyclization and/or rearrangement of linear isoprenoid diphosphates into cyclic terpenoids [12, 13]. Given the vast variety of terpenoids, it is anticipated that the exact architecture of the active site cavity is strictly defined in specific synthases [13]. This is particularly true in the case of sesquiterpene synthases that catalyze reactions to yield a myriad of structurally diverse C15-hydrocarbons using the same substrate FPP [14]. Due to the complexity of the catalytic reaction cascade, rational engineering of terpenoid synthases to change product specificity or to enhance activity remains a challenge [12, 15]. However, sequence comparisons among closely related but functionally different enzymes combined with mutagenesis to change synthase performance have been proved to be effective in quite a few cases [12, 16, 17].

Sesquiterpene ß-elemene, a major component in Chinese medicinal herb, *Curcuma wenyujin* T. H. Chen et C. Ling, displays antitumor activity against a variety of tumor types [18] and has been clinically administrated to tumor treatments. The sesquiterpene synthase for ß-elemene has not been identified from *Curcuma wenyujin* and other related plants. Instead, it is generally considered that ß-elemene is transformed from germacrene A, which is synthesized by germacrene A synthase (GAS). Under heating or acidic conditions, germacrene A is automatically converted to ß-elemene via one-step intramolecular rearrangement. The in vitro transformation occurs even at room temperature [19]. Therefore, synthesis of germacrene A by dedicated synthases is a promising approach to efficiently produce ß-elemene, which would greatly lower its cost of production. Hu et al. have successfully established a production platform to produce germacrene A by expressing a GAS (LTC2) of *Lactuca sativa* in a yeast chassis strain that has been optimized with isoprenoid production [20]. Germacrene A production was further increased by introduction of one more copy of tHMGR1 expression cassette and expression of fused LTC2 and ERG20 with a final titer of 190.7 mg/L in shake flasks [20]. Moreover, Zhang et al. [21] have reported the development of coupling techniques for thermal conversion of germacrene A to ß-elemene, which reduces the production cost of β-elemene to 0.15% of that from plant extraction.

**Fig. 1:**
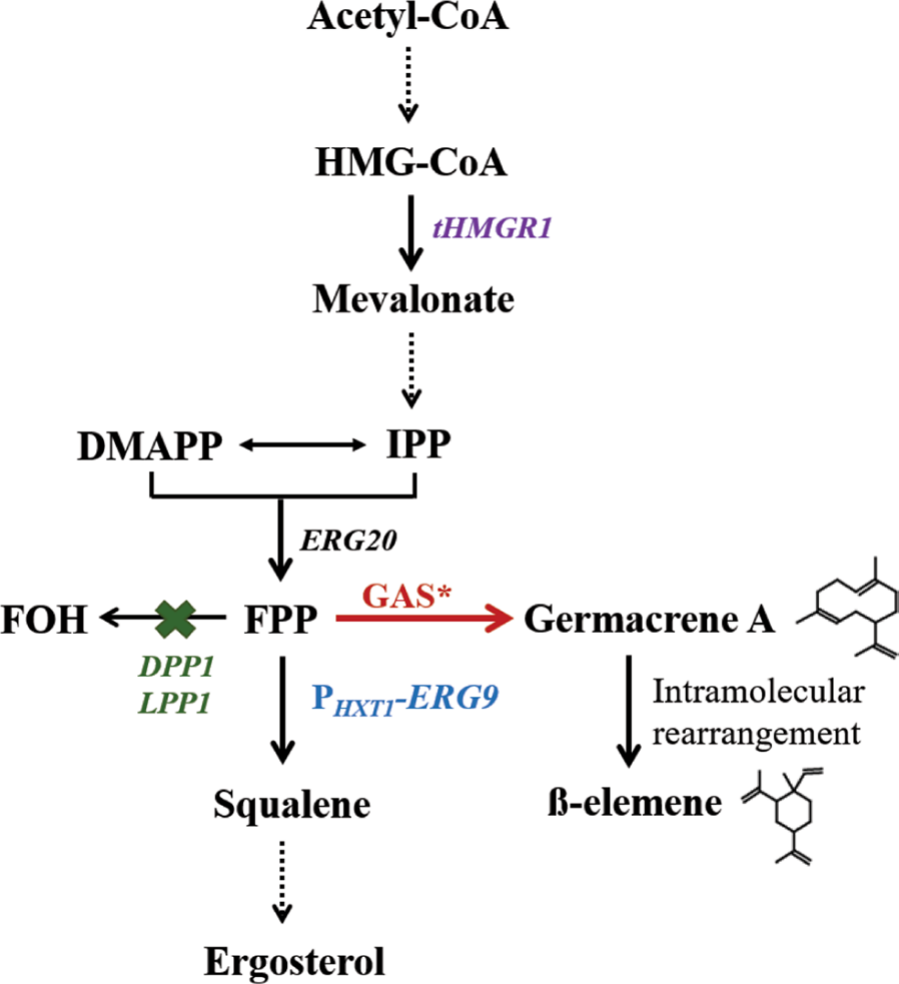
Schematic overview of germacrene A production in *S. cerevisiae*. To improve germacrene A production, GAS (marked in red) screening and site-directed mutagenesis (represented with an asterisk) were performed, the *tHMGR1* gene (marked in purple) was overexpressed, the *LPP1* and *DPP1* genes (marked in green) were deleted, and the *ERG9* gene (marked in blue) was downregulated by replacing its native promoter with the promoter of *HXT1*. Acetyl-CoA: acetyl coenzyme A, HMG-CoA: 3-hydroxy-3-methylglutaryl-CoA, *tHMGR1*: truncated HMG-CoA reductase gene, IPP: isopentenyl pyrophosphate, DMAPP: dimethylallyl pyrophosphate, *ERG20*: farnesyl diphosphate synthase gene, FPP: farnesyl diphosphate, FOH: farnesol, *ERG9*: squalene synthase gene, GAS: germacrene A synthase. Solid arrows represent one-step transformation while dashed arrows represent transformation comprising multiple intermediate steps

In this study, we aimed to improve germacrene A biosynthesis in engineered *S. cerevisiae* (Fig. 1). We first performed screening with several identified or putative GASs from different sources, and found that a cyanobacterial synthase, *Av*GAS, showed the best performance on germacrene A synthesis. Known strategies of engineering the MVA pathway including expression of tHMGR1 using a constitutive promoter and repression of squalene synthesis pathway via replacing the *EGR9* promoter with the *HXT1* promoter, further improved germacrene A production. Site-directed mutagenesis of *Av*GAS was simultaneously performed and *Av*GAS F23W mutant was found to enable recombinant yeast strains to produce 309.8 mg/L germacrene A in shake-flask cultivation.

## Results

### Screening GASs with better performance on germacrene A synthesis

**Fig. 2:**
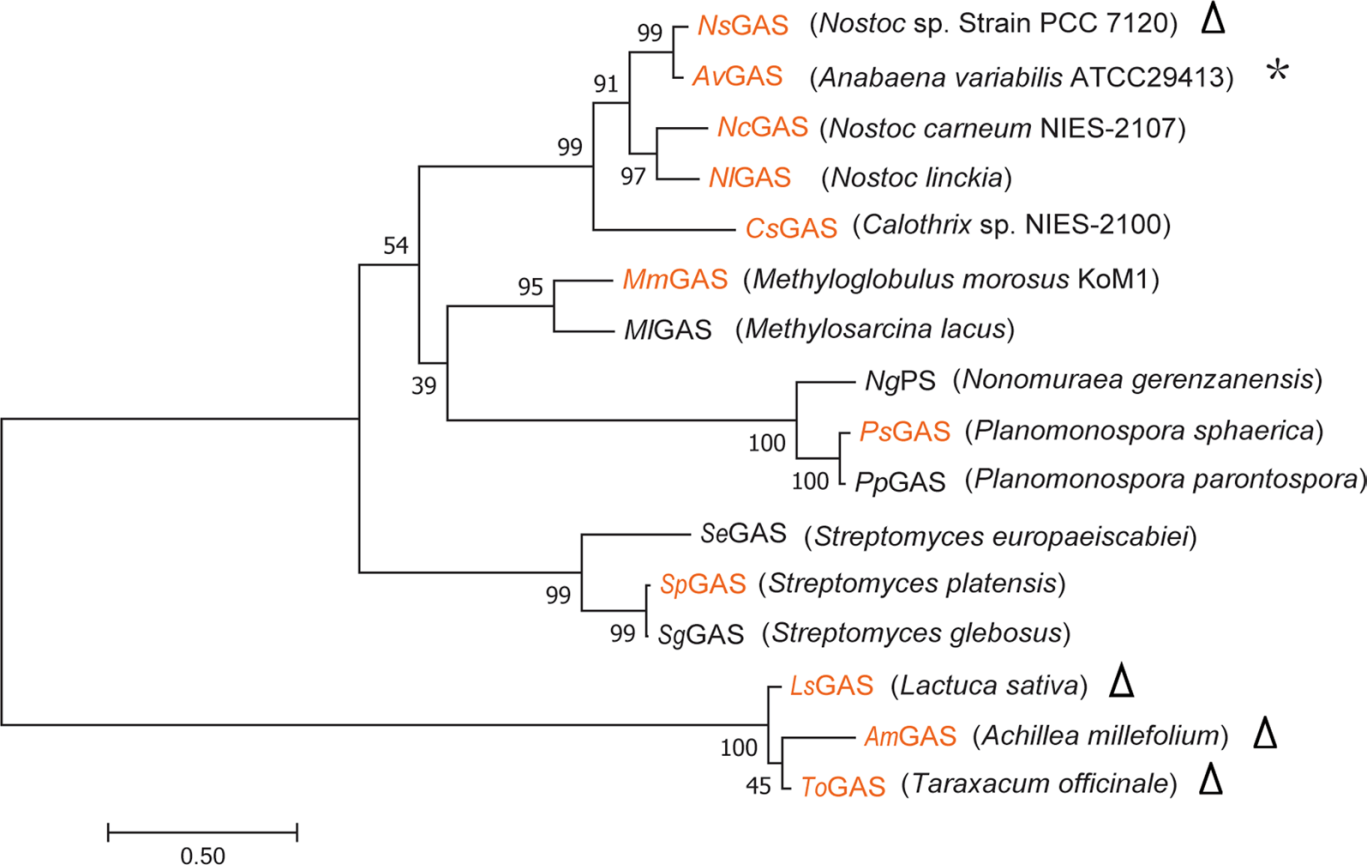
Phylogenetic analyses of the putative GASs used for enzyme screening and their orthologues. Previously known GASs were indicated by triangles. *Av*GAS identified in this study with the best performance on germacrene A production was indicated with an asterisk. The ten synthases applied for screening in this study were colored orange. The protein sequences of putative GASs or their orthologues were retrieved from NCBI or Uniprot databases with accession or entry numbers as follows: *NsGAS* (NCBI accession NO. WP_010998816), *Am*GAS (NCBI accession NO. AGD80135), *To*GAS (NCBI accession NO. ALY05868), *Ls*GAS (LTC2, NCBI accession NO. AAM11627), *Av*GAS (UniProt entry NO. Q3MBN2), *NcGAS* (UniProt entry NO. A0A1Z4HUB4), *Mm*GAS (UniProt entry NO. V5BIL8), *Cs*GAS (UniProt entry NO. A0A1Z4H7M3), *Sp*GAS (UniProt entry NO. A0A1Y2N8F8), *Ps*GAS (UniProt entry NO. A0A171DER6), *Nl*GAS (NCBI accession NO. WP_190654468), *Ml*GAS (NCBI accession NO. WP_024299200), *Sg*GAS (NCBI accession NO. WP_190143472), *Se*GAS (NCBI accession NO. WP_060893906), *Pp*GAS (NCBI accession NO. WP_189237389), *Ng*PS (Pentalenene synthase, NCBI accession NO. SBO99268)

**Fig. 3:**
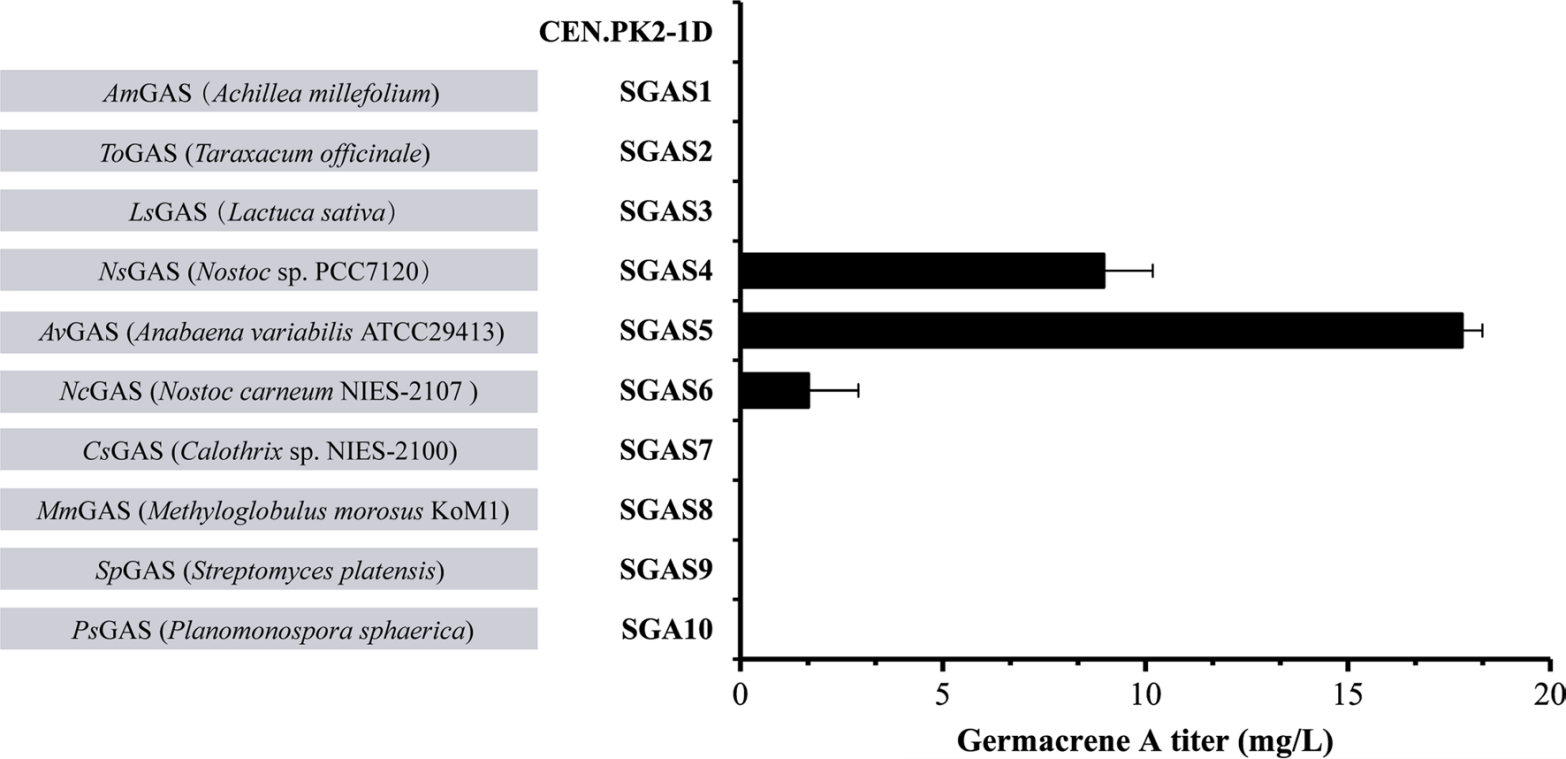
Screening GASs with better performance on germacrene A synthesis. Expression of the synthases from various sources were performed in the parent *S. cerevisiae* CEN.PK2-1D strain. Values represent the means of three biological replicates. Error bars are the standard deviations from these replicates

GAS catalyzes the reaction to synthesize germacrene A from FPP. To increase germacrene A production, several identified and putative GASs from different species were expressed in *S. cerevisiae* (Fig. 2). Germacrene A synthesis in these recombinant strains wherein FPP was solely derived from the endogenous MVA pathway, was evaluated. Since germacrene A was completely converted to ß-elemene at high temperature during gas chromatography analysis, ß-elemene was applied as a standard for detection and quantification of germacrene A extracted from yeast cultures (Additional file 1: Figure S1). Preliminary screening was performed with three GASs previously identified from plants including *Achillea millefolium* [22], *Taraxacum officinale* [23] and *Lactuca sativa* [20], and one identified from the bacterium *Nostoc* sp. PCC 7120 [24]. As shown in Fig. 3, expression of the three plant-derived GASs in *S. cerevisiae* led to no detectable germacrene A production compared with the parent *S. cerevisiae* CEN.PK2-1D strain. In contrast, expression of the cyanobacterial GAS (*Ns*GAS) resulted in a significant production of germacrene A. We therefore chose this synthase for the next round of screening.

Protein blast was performed in UniProt database with *Ns*GAS as a query and bacterial orthologues sharing various sequence identities with *Ns*GAS were retrieved. Six candidates were selected for further analysis, including putative GASs of *Anabaena variabilis* ATCC29413 (*Av*GAS, identity 93.2%), *Nostoc carneum* NIES-2107 (*Nc*GAS, identity 70.8%), *Calothrix* sp. NIES-2100 (*Cs*GAS, identity 55%), *Methyloglobulus morosus* KoM1 (*Mm*GAS, identity 38.6%), *Streptomyces platensis* (*Sp*GAS, identity 31%) and *Planomonospora sphaerica* (*Ps*GAS, identity 27%) (Fig. 2; Additional file 1: Figure S2). Only two synthases, *Nc*GAS and *Av*GAS, conferred germacrene A-producing capability on yeast cells, indicating that these two synthases are effective GASs. While *Nc*GAS expression led to much less ß-elemene synthesis than *Ns*GAS, expression of *Av*GAS resulted in a twofold increase in ß-elemene production reaching a titer of 17.8 mg/L in the corresponding yeast strain SGAS5. These results indicate that *Av*GAS of *A. variabilis* ATCC29413 exhibited the best performance on germacrene A production among the screened synthases when expressed in yeast, and was therefore selected for further engineering study.

### Engineering the MVA pathway to improve germacrene A biosynthesis

**Fig. 4:**
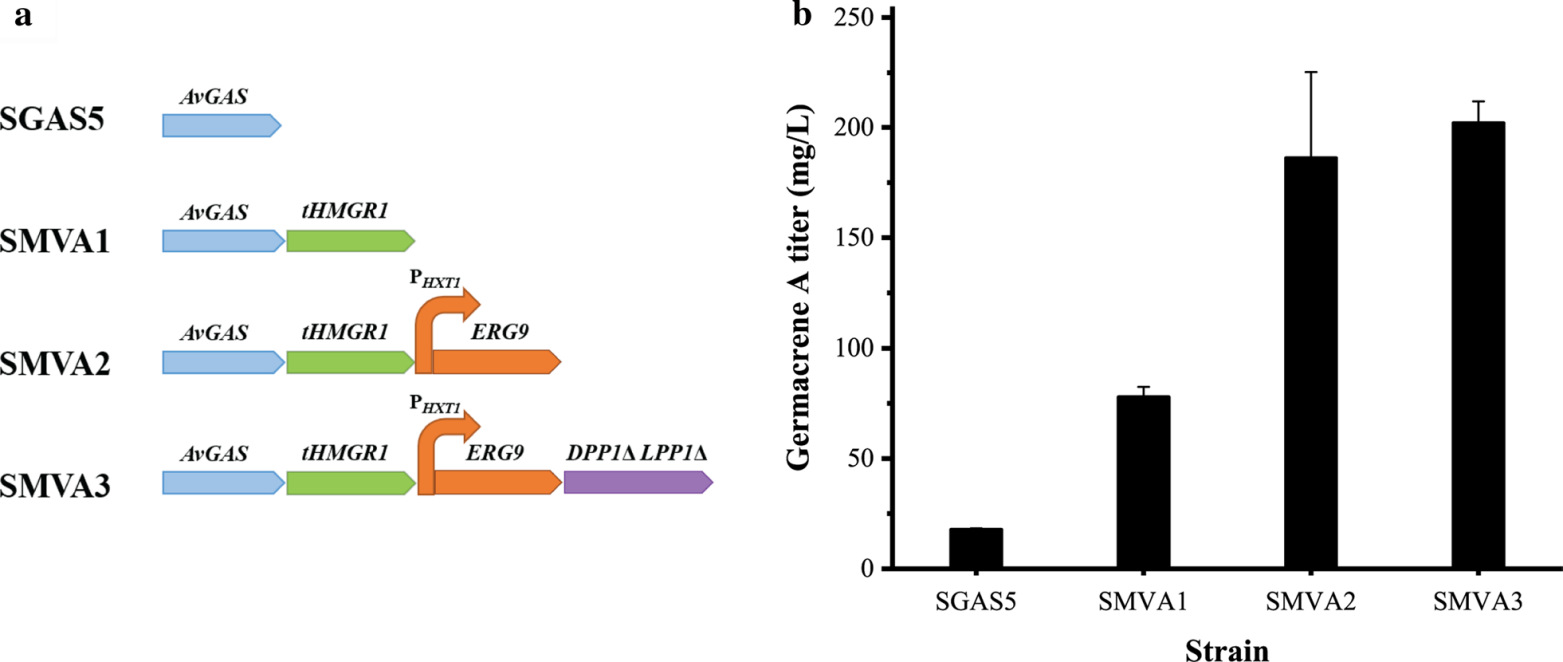
Germacrene A production was enhanced by increasing the precursor FPP availability in yeast. Strategies include overexpression of tHMGR1, repression of *ERG9* expression by replacing its native promoter with *HXT1* promoter, and deletion of *DPP1* and *LPP1*. Values represent the means of three biological replicates. Error bars are the standard deviations from these replicates

FPP from the yeast MVA pathway is the direct precursor for sesquiterpene synthesis. To improve sesquiterpene production, it is necessary to enhance FPP availability. Frequently used strategies to metabolically engineer yeast chassis to increase FPP supply include overexpression of the rate-limiting enzyme tHMGR1 of the MVA pathway and repression of the competing squalene synthesis pathway and farnesol synthesis pathway [3–6]. Overexpression of tHMGR1 in strain SGAS5 resulted in strain SMVA1, which showed a 4.4-fold increase in germacrene A production, reaching a titer of 77.8 mg/L (Fig. 4). Repressing squalene synthesis in strain SMVA1 by replacing the *ERG9* promoter with the *HXT1* promoter [25, 26] was found to further increase germacrene A production up to 186.2 mg/L in the resultant SMVA2 strain (Fig. 4). Along with the enhanced germacrene A production, farnesol synthesis was also significantly increased due to the enhanced FPP supply (Additional file 1: Figure S3). Two phosphatase-encoding genes, *DPP1* and *LPP1*, involved in farnesol synthesis were therefore deleted in strain SMVA2 to obtain the SMVA3 strain. Whereas farnesol accumulation was decreased by 24.6%, germacrene A production in SMVA3 was not significantly increased (Fig. 4; Additional file 1: Figure S3).

### Engineering *Av*GAS for improved germacrene A biosynthesis via site-directed mutagenesis

**Fig. 5:**
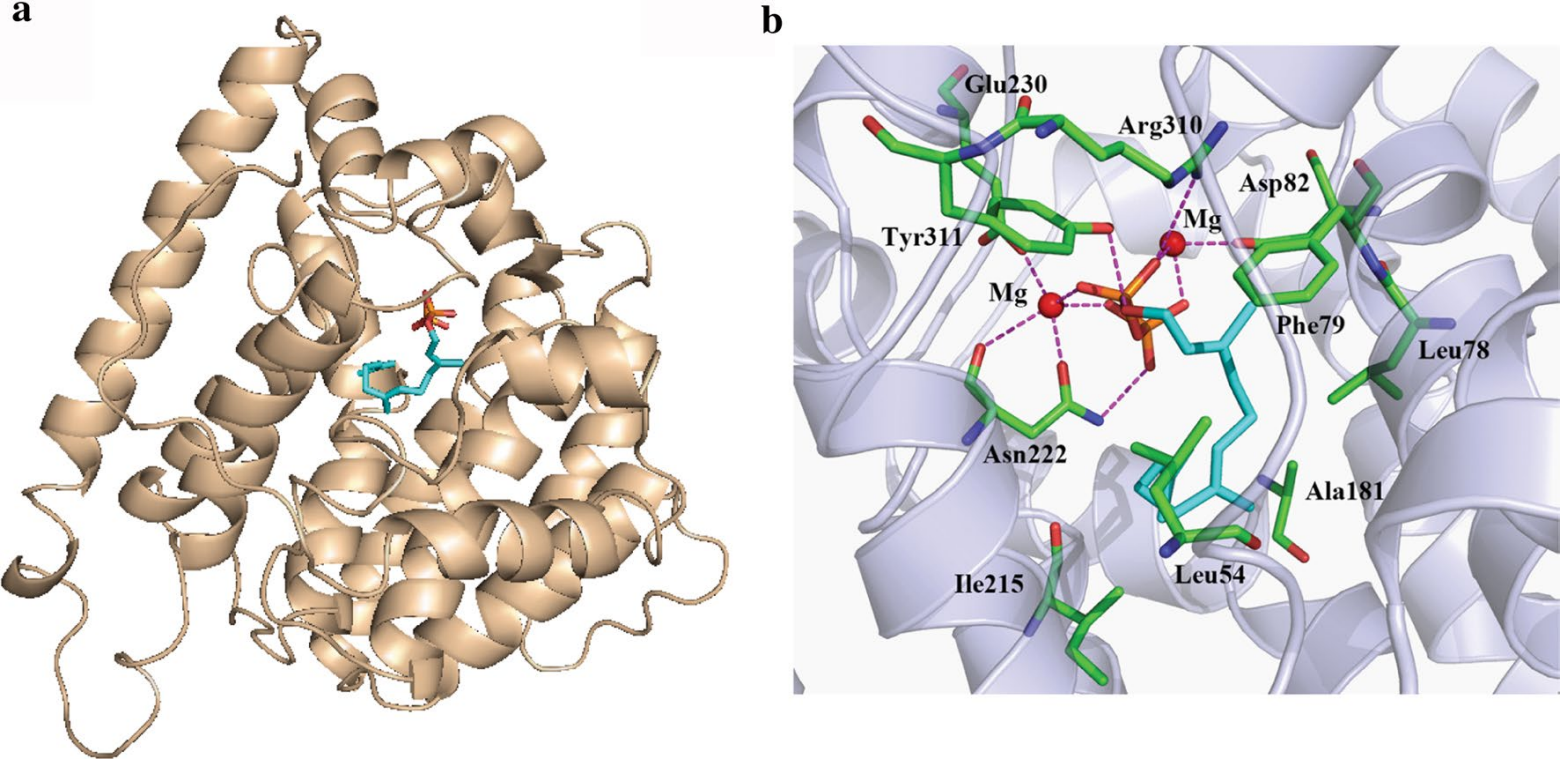
Modelled structure of *Av*GAS (**a**) and molecular docking simulation with substrate FPP (**b**). FPP is represented as cyan sticks and amino acids as indicted are shown as green sticks

While enhanced FPP supply resulted in not only increased production of the target product, but also accumulation of the byproduct farnesol, implying that FPP flux towards germacrene A synthesis is becoming a limiting step. We therefore attempted to engineer *Av*GAS to improve its catalytic capability of synthesizing germacrene A via site-directed mutagenesis. Homology modelling was first performed with the resolved structure of a bacterial 1,8-cineole synthase in complex with a FPP analogue (PDB ID: 5NX6) [14] as a template. As shown in Fig. 5a, like most bacterial terpene synthases, modelled *Av*GAS adopts a common α-helical fold with a single catalytic domain, but lacks the additional N-terminal α-barrel domain characteristic of plant enzymes [27]. Two conserved regions within the active site, the aspartate-rich (DDXX(X)(D,E)) motif and the NSE (NDXXSXX(R,K)(E,D)) triad [14, 28], required for binding catalytically essential Mg^2+^ ions, were also present in *Av*GAS (Additional file 1: Figure S4).

### Site-directed mutagenesis of residues located in the catalytic pocket of AvGAS severely compromised germacrene A biosynthesis

**Fig. 6:**
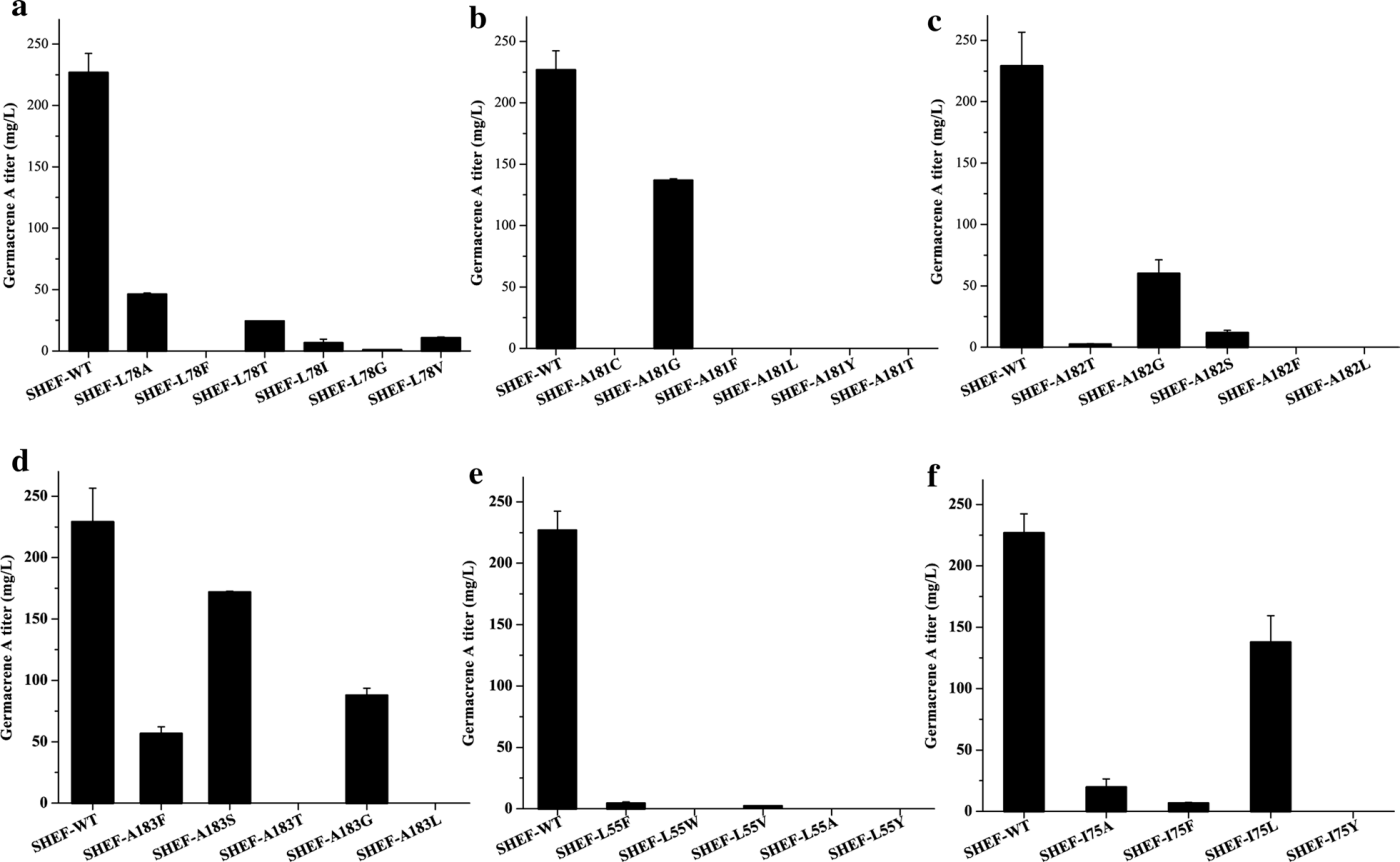
Production of germacrene A of yeast strains expressing *Av*GAS mutants carrying site-directed mutagenesis of residues located within the active site cavity. Mutations were performed on residues including L78 (**a**), A181 (**b**), A182 (**c**), A183 (**d**), L55 (**e**), and I75 (**f**). Values represent the means of three biological replicates. Error bars are the standard deviations from these replicates

Based on the modelled structure of *Av*GAS, molecular docking simulation of *Av*GAS with substrate FPP was performed (Fig. 5b). In addition to coordination by Mg^2+^ ions, the pyrophosphate moiety of FPP makes interactions with residues including Arg310, Asn222, and Tyr311 (Fig. 5b). The hydrophobic FPP carbon chain interacts with several residues including Leu54, Leu78, Ala181, and Ile 215 within the substrate binding pocket via Van der Waals' force. While residues involved in binding the pyrophosphate moiety of FPP are evolutionarily conserved in terpene synthases, it is the hydrophobic cavity essentially mediates the sequential FPP carbon chain cyclization [14], We therefore attempted to perform mutations on residues Leu78 and Ala181 as well as residues Leu55, Ala182, Ala183, and Ile75 that are also located within the substrate binding cavity. Strain SHEF was first constructed with CEN.PK2-1D as the parent strain for evaluation of various *Av*GAS site-directed mutants, wherein tHMGR1 was overexpressed, the *EGR9* promoter was replaced with the *HXT1* promoter, and *LPP1* and *DPP1* were deleted. The wild type (WT) and resultant mutants of *Av*GAS were expressed in the SHEF strain. Whereas substitution of Leu78 with Ala, Thr, Ile, Val, or Gly led to significantly decreased germacrene A production, its replacement with Phe completely abolished production of the target product (Fig. 6a). Similarly, mutations of Ala181 (to Cys, Gly, Phe, Leu, Tyr, or Thr), Ala182 (to Thr, Gly, Ser, Phe or Leu), Ala183 (to Phe, Ser, Thr, Gly or Leu), Leu55 (to Phe, Trp, Val, Ala or Tyr), and Ile75 (to Ala, Leu, Tyr or Phe) all resulted in markedly decreased or undetectable germacrene A accumulation (Fig. 6b–f). These results indicate that residues located in the catalytic cavity of *Av*GAS play key roles in biosynthesis of germacrene A.

### Expression of the AvGAS mutants F23W and F23V improved germacrene A biosynthesis

**Fig. 7:**
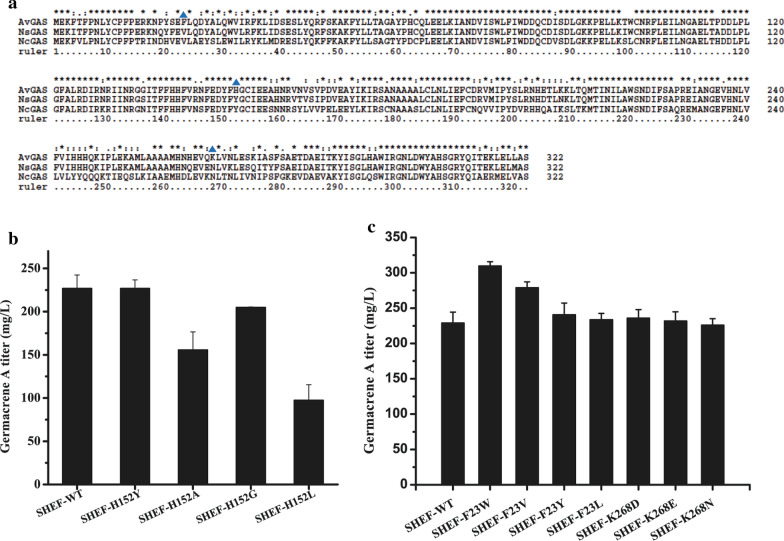
Production of germacrene A of yeast strains expressing *Av*GAS mutants carrying site-directed mutagenesis of residues Phe23, His152, and Lys268. **a** Sequence alignment of *Av*GAS with its two highly identical orthologues *Ns*GAS and *Nc*GAs. Phe23, His152, and Lys268 are indicated by blue triangles. **b**, **c** The effect of site-directed mutations of His152 (**b**), Phe23 and K268 (**c**) on germacrene A production-producing capability of *Av*GAS when expressed in yeast. Values represent the means of five biological replicates. Error bars are the standard deviations from these replicates

Since mutation of residues within the catalytic cavity of *Av*GAS failed to produce a mutant enzyme with improved capability in germacrene A biosynthesis, we next analyzed the impact of mutations of some “variable” residues whose change may contribute to enzyme activity. *Av*GAS and its two orthologues with relatively higher identities, *Ns*GAS and *Nc*GAS, are assumed to display significant different enzymatic activities since their expression in yeast led to different germacrene A accumulation as described above. Sequence alignment of *Av*GAS with *Ns*GAS and *Nc*GAS revealed that three residues located far from the active site cavity were identical at the corresponding positions for *Ns*GAS and *Nc*GAS (Val23, Tyr152, and Asn268), but distinct in *Av*GAS (Phe23, His152 and Lys268) (Fig. 7a). To see whether changes in these residues could have an effect on the enzymatic activity of *Av*GAS, Phe23 was replaced with Val, Tyr, Leu, and Trp, respectively, His152 was changed to Tyr, Ala, Gly, and Leu, respectively, and Lys268 was substituted with Arg, Asp, and Glu, respectively. Unlike mutations within the active site cavity, none of these mutations abolished germacrene A synthesis (Fig. 7b, c). Expression of a majority of these mutants allowed yeast cells to accumulate decreased or similar levels of germacrene A compared to cells expressing WT *Av*GAS. Notably, two yeast strains expressing *Av*GAS-F23W and *Av*GAS-F23V displayed markedly improved germacrene A production (35.2% and 21.8% increase, respectively). The production titer of germacrene A in strains SHEF-F23W and SHEF-F23V reached 309.8 mg/L and 278.9 mg/L, respectively. The increase in product accumulation is not due to improved cell growth since SHEF-F23W and SHEF-F23V showed similar biomass accumulation with other *Av*GAS mutant strains (Additional file 1: Table S1).

## Discussion

The sesquiterpene germacrene A is a direct precursor of ß-elemene that exhibits therapeutic efficacy against various types of tumors [18]. In this study, we combined GAS screening, site-directed mutagenesis engineering, and metabolic engineering of yeast chassis to improve germacrene A production.

In a previous study to increase germacrene A production in yeast, Hu et al. [20] has evaluated the capability of two GASs from plants and selected LTC2 of *Lactuca sativa* to achieve a relatively high production yield. Here, in addition to LTC2 (here termed *Ls*GAS), we also included nine more known or putative GASs from plants or bacteria for evaluation. When expressed in a *S. cerevisiae* strain without the MVA pathway engineering, only three cyanobacterial synthases, *Ns*GAS, *Nc*GAS, and *Av*GAS, showed capability of synthesizing germacrene A. The inability of LTC2 (*Ls*GAS) to catalyze the detectable germacrene A formation in the present study may result from the use of yeast strain with a different genetic background. Hu et al. employed a parent strain with overexpression of tHMGR1 and ERG20, repression of *ERG9*, and deletion of *DPP1* and *LPP1*, wherein the MVA flux and FPP supply was significantly enhanced. LTC2 (*Ls*GAS) and *Tm*GAS were indeed found to successfully mediate germacrene A production, respectively, in an engineered yeast strain with tHMGR1 overexpression and *ERG9* repression (Additional file 1: Figure S5). Nonetheless, the detected target product synthesis was still markedly lower than those resultant from the respective expression of *Ns*GAS, *Nc*GAS, and *Av*GAS (Additional file 1: Figure S5), supporting that these three cyanobacterial synthases exhibited much better performance in germacrene A synthesis compared to LTC2 (*Ls*GAS). Our result is consistent with the previous report that bacterial terpene synthase outperforms the orthologous plant enzyme: bLinS of *Streptomyces clavuligerus* produced about 300-fold more linalool than RLinS_Aa of *Artemisia annua* [14]. Structural analyses revealed that, unlike plant enzymes comprising the N-terminal α-barrel domain and the C-terminal α-helix domain, the majority of bacterial terpene sesquiterpene synthases contain only the α-helices constituting a single catalytic domain. In addition to higher catalytic activity, the smaller size and simplified structure of bacterial synthases might facilitate its expression in yeast and hence contribute to the catalyzed formation of target product. These possibilities definitely warrants further investigation.

Increasing precursor supply is crucial in ensuring high-level target product synthesis. When some routinely used strategies were applied to enhance precursor FPP supply in yeast, a marked increase in germacrene A production was observed. However, we noticed that the byproduct farnesol also increased with the target product. Although the two phosphatase encoding genes *DPP1* and *LPP1* were deleted, farnesol accumulation was not completely eliminated. It is highly probable that other as yet to be identified phosphatases exist to compensate for the loss of DPP1 and LPP1, such as PHO8, APP1, and PAH1 [29, 30]. Despite the increased farnesol production, Hu et al. reported that introduction of one additional copy of tHMGR1 expression cassette led to a further 1.5-fold increase in germacrene A production, indicating that directing more flux into the MVA pathway to enhance the precursor pool is beneficial for germacrene A accumulation. We also noticed that farnesol production in yeast strain SHEF expressing *Av*GAS is markedly decreased compared to that expressing plant *Ls*GAS, and a further reduction was observed in SHEF strain expressing *Av*GAS F23W mutant, suggesting that efficient GAS contribute to shifting the flux from farnesol synthesis to germacrene A synthesis (Additional file 1: Figure S6). Furthermore, it was observed that germacrene A was also trapped intracellularly, when we broke SHEF cells, and found that germacrene A accumulates in intracellular fractions (Additional file 1: Figure S7). Future studies on identification of the membrane efflux transporters which could efficiently export germacrene A to culture supernatant might contribute to decreasing intracellular accumulation and therefore enhancing final extracellular yield.

Compared to the various already known strategies to effectively engineering the *S. cerevisiae* MAV pathway, terpene synthases responsible for the cyclization of linear isoprenoid diphosphates into cyclic terpenoids are intractable to be engineered [12, 15]. The difficulty mainly lies in the complexity of the reaction cascade occurring within the catalytic cavity of the synthase and the lack of high-throughput screening method. Indeed, repeated attempts to target residues within the active site cavity of *Av*GAS to improve its activity failed, as demonstrated by the undetectable or markedly decreased germacrene A production with the relevant *Av*GAS mutants (Fig. 6). It is thus highly probable that most, if not all, residues along the cavity may directly or indirectly participate in the sequential cyclization of FPP and thus play important or essential roles in the catalytic process. Given that the same substrate FPP can be transformed into a myriad of various sesquiterpenes by dedicated synthases, one can speculate that the exact shape and size as well as the hydrophobicity of the active site cavity of the respective synthase is vitally essential for specific product formation. The observation that variation of Ala181 to Gly with a similar size decreased but not abolished germacrene A production, whereas its replacement with larger residues including Cys, Phe, Leu, Tyr or Thr resulted in undetectable production of germacrene A, suggests that maintenance of the precise configuration of the active site cavity is crucial for the efficient carryon of the reaction cascade. Similar phenomenon was also observed for Ala182 and Ile75 wherein mutation of Ala to Gly and Ile to Leu resulted in the mildest impact on germacrene A yield among all the tested mutations. However, exceptions to the above scenario also exist. Variation of Leu78 to Ile with similar size and hydrophobicity resulted in a more severe impact on germacrene A production compared to replacement by Ala with smaller size and lower hydrophobicity. Moreover, while A183G was more efficient in product synthesis than A183F, change of Ala to hydrophilic Ser (A183S) outperformed both A183G and A183F. This discrepancy further implicates that any subtle perturbation of the precise configuration of the active site cavity or the intricate interaction network between enzyme and substrate might exert a significant disturbing effect on the enzyme activity. Possibility also can not be excluded that the relevant mutations just destroy the overall structure of *Av*GAS and thus reduced the solubility of the expressed synthase in yeast cells. Of the three cyanobacterial germacrene A synthases, *Av*GAS showed the best performance in germacrene A biosynthesis when expressed in yeast. Whereas the reason for the higher capability of *Av*GAS as compared to the other two highly identical orthologues is not clear at present, sequence alignment did reveal various residues in *Av*GAS that are distinct from its corresponding orthologues. Three such residues including His152, Phe23, and Lys268 were selected for site-directed mutagenesis. Whereas quite a few mutants did not affect germacrene production, two resultant mutants *Av*GAS-F23W and *Av*GAS-F23V enabled improved germacrene A production compared with WT *Av*GAS, resulting in an increase of 35.2% and 21.8% in production yield, respectively. In contrast, mutants H152A, H152L, and H152G exhibited decreased capability in germacrene A production. These substitutions most probably affected the hydrophilicity of His152 site and thus exerted a negative effect on the performance of *Av*GAS. Considering that Phe23 is located on the lateral surface of *Av*GAS where is relatively far way from the active site cavity (Additional file 1: Figure S8), it is speculated that these two mutations exert their effect via interacting with its proximal residues which then indirectly affects substrate accommodation or product release and thus germacrene A synthesis. Possibility can not be excluded that these two beneficial mutations of Phe23 facilitated functional *Av*GAS soluble expression in yeast cells, which contributes to the observed increase in product yield. It can be expected that more strategies such as introduction of one more copy of tHMGR1, overexpression of ERG20, and appropriate fusion of ERG20 with the cyanobacterial synthase, which were employed in previous biosynthesis of germacrene A [20], would further increase the yield to a large margin.

## Materials

### Strains and culture conditions

*Escherichia coli* DH5α was used for routine plasmid construction and was cultivated at 37 °C in Luria–Bertani (LB) medium. When necessary, 100 µg/mL of ampicillin was supplemented in LB medium. Parent strain *S. cerevisiae* CEN.PK2-1D was cultured at 28 °C in yeast extract peptone dextrose (YPD) medium. Engineered yeast strains were cultivated in synthetic complete (SC) drop-out media, and 50 µg/mL of leucine, histidine, tryptophan, uracil, or 40 µg/mL of geneticin (G418) were individually or simultaneously added when necessary.

### Plasmids and strains construction

All the plasmids and strains used in this study were listed in Table 1. For GAS screening, totally ten identified or putative GAS encoding genes or cDNA sequences including *NsGAS* (NCBI accession NO. WP_010998816), *Am*GAS (NCBI accession NO. AGD80135), *To*GAS (NCBI accession NO. ALY05868), *Ls*GAS (NCBI accession NO. AAM11627), *Av*GAS (UniProt entry NO. Q3MBN2), *NcGAS* (UniProt entry NO. A0A1Z4HUB4), *Mm*GAS (UniProt entry NO. V5BIL8), *Cs*GAS (UniProt entry NO. A0A1Z4H7M3), *Sp*GAS (UniProt entry NO. A0A1Y2N8F8), and *Ps*GAS (UniProt entry NO. A0A171DER6), were codon-optimized according to the *S. cerevisiae* codon bias and synthesized by GenScript Biotech Corporation (Suzhou, China). These *GAS* gene fragments were ligated into the pRS425 plasmid carrying the *GAL1* promoter and the *ADH1* terminator that were both amplified from *S. cerevisiae* W303 genomic DNA. The resultant plasmids for expression of GASs were individually transformed into *S. cerevisiae* CEN.PK2-1D using the PEG/LiAc method [31]. To overexpress tHMGR1, a truncated version of *HMGR1* encoding fragment [7] was amplified with *S. cerevisiae* CEN.PK2-1D genomic DNAas template, and ligated into the pRS306 plasmid that harbors the *TPI1* promoter and the *CYC1* terminator. The resultant plasmid pRS306*-tHMGR1* was transformed into strain SGAS5 to generate SMVA1. The *HXT1* promoter and the loxp-*KanMX-*loxp cassette were amplified from *S. cerevisiae* CEN.PK2-1D genomic DNA and plasmid pUG6 [32], respectively, and fused together via overlap extension PCR [33]. A pair of primers containing 70-bp nucleotide fragments upstream and downstream of the native *ERG9* promoter, respectively, were used to amplify the fused fragment, to construct the *ERG9* promoter replacement cassette. This cassette was subsequently integrated into strain SMVA1, resulting in strain SMVA2 wherein the native *ERG9* promoter was replaced by the *HXT1* promoter. To delete *DPP1*, a pair of primers containing 70-bp nucleotide fragments upstream and downstream of the *DPP1* gene was used to amplify the loxp-*KanMX-*loxp cassette to construct *DPP1* gene deletion cassette. This cassette was integrated into SMVA2 wherein the *KanMX* selection marker was removed ahead of time. The deletion cassette of *LPP1* was constructed similarly, and integrated into strain SMVA2 with deleted *DPP1*, resulting in strain SMVA3 wherein *DPP1* and *LPP1* were both deleted. To construct strain SHEF, pRS306*-tHMGR1*, *ERG9* promoter replacement cassette, and *DPP1* and *LPP1* deletion cassettes were integrated into *S. cerevisiae* CEN.PK2-1D in succession using the same procedures as described above. To generate *AvGAS* fragments with specific site-directed mutations, overlap-extension PCR was performed with pRS425-*AvGAS* as template. The mutated fragments were ligated into the pRS425 plasmid carrying the *GAL1* promoter and the *ADH1* terminator. DNA sequencing was performed to confirm that each mutagenesis occurred as expected. The pRS425-derived plasmids containing the expression cassette of WT and various site-specific mutated *AvGAS* genes were transformed into strain SHEF, respectively.

**Table 1:** Plasmids and strains used in this study

Plasmids and strains	Description	Source or reference
Plasmids		
pRS306	Integrative plasmid, *URA3*, *Amp*^*r*^	Laboratory stock
pRS306*-tHMGR1*	*URA3*, pRS306*-*P_*TPI1*_*-tHMGR1*-T_*CYC1*_	This study
pUG6	*loxp-KanMX-loxp*, *Amp*^*r*^	Laboratory stock
pRS425	2μ ori, *LEU2*, *Amp*^*r*^	Laboratory stock
pRS425-*NsGAS*	*LEU2*, pRS425-P_*GAL1*_-*NsGAS*-T_*ADH1*_	This study
pRS425-*AmGAS*	*LEU2*, pRS425-P_*GAL1*_-*AmGAS*-T_*ADH1*_	This study
pRS425-*ToGAS*	*LEU2*, pRS425-P_*GAL1*_-*ToGAS*-T_*ADH1*_	This study
pRS425-*LsGAS*	*LEU2*, pRS425-P_*GAL1*_-*LsGAS*-T_*ADH1*_	This study
pRS425-*AvGAS*	*LEU2*, pRS425-P_*GAL1*_-*AvGAS*-T_*ADH1*_	This study
pRS425-*NcGAS*	*LEU2*, pRS425-P_*GAL1*_-*NcGAS*-T_*ADH1*_	This study
pRS425-*CsGAS*	*LEU2*, pRS425-P_*GAL1*_-*CsGAS*-T_*ADH1*_	This study
pRS425-*MmGAS*	*LEU2*, pRS425-P_*GAL1*_-*MmGAS*-T_*ADH1*_	This study
pRS425-*SpGAS*	*LEU2*, pRS425-P_*GAL1*_-*SpGAS*-T_*ADH1*_	This study
pRS425-*PsGAS*	*LEU2*, pRS425-P_*GAL1*_-*PsGAS*-T_*ADH1*_	This study
pRS425-*AvGAS-L78A*	*LEU2*, pRS425-P_*GAL1*_- *AvGAS-L78A*-T_*ADH1*_	This study
pRS425- *AvGAS-L78F*	*LEU2*, pRS425-P_*GAL1*_- *AvGAS-L78F*-T_*ADH1*_	This study
pRS425- *AvGAS-L78T*	*LEU2*, pRS425-P_*GAL1*_- *AvGAS-L78T*-T_*ADH1*_	This study
pRS425-*AvGAS-L78I*	*LEU2*, pRS425-P_*GAL1*_- *AvGAS-L78I*-T_*ADH1*_	This study
pRS425-*AvGAS-L78G*	*LEU2*, pRS425-P_*GAL1*_- *AvGAS-L78G*-T_*ADH1*_	This study
pRS425-*AvGAS-L78V*	*LEU2*, pRS425-P_*GAL1*_- *AvGAS-L78V*-T_*ADH1*_	This study
pRS425-*AvGAS-A181C*	*LEU2*, pRS425-P_*GAL1*_- *AvGAS-A181C*-T_*ADH1*_	This study
pRS425-*AvGAS-A181G*	*LEU2*, pRS425-P_*GAL1*_- *AvGAS-A181G*-T_*ADH1*_	This study
pRS425-*AvGAS-A181F*	*LEU2*, pRS425-P_*GAL1*_- *AvGAS-A181F*-T_*ADH1*_	This study
pRS425-*AvGAS-A181L*	*LEU2*, pRS425-P_*GAL1*_- *AvGAS-A181L*-T_*ADH1*_	This study
pRS425-*AvGAS-A181Y*	*LEU2*, pRS425-P_*GAL1*_- *AvGAS-A181Y*-T_*ADH1*_	This study
pRS425-*AvGAS-A181T*	*LEU2*, pRS425-P_*GAL1*_- *AvGAS-A181T*-T_*ADH1*_	This study
pRS425-*AvGAS-A182T*	*LEU2*, pRS425-P_*GAL1*_- *AvGAS-A182T*-T_*ADH1*_	This study
pRS425-*AvGAS-A182G*	*LEU2*, pRS425-P_*GAL1*_- *AvGAS-A182G*-T_*ADH1*_	This study
pRS425-*AvGAS-A182S*	*LEU2*, pRS425-P_*GAL1*_- *AvGAS-A182S*-T_*ADH1*_	This study
pRS425-*AvGAS-A182F*	*LEU2*, pRS425-P_*GAL1*_- *AvGAS-A182F*-T_*ADH1*_	This study
pRS425-*AvGAS-A182L*	*LEU2*, pRS425-P_*GAL1*_- *AvGAS-A182L*-T_*ADH1*_	This study
pRS425-*AvGAS-A183F*	*LEU2*, pRS425-P_*GAL1*_- *AvGAS-A183F*-T_*ADH1*_	This study
pRS425-*AvGAS-A183S*	*LEU2*, pRS425-P_*GAL1*_- *AvGAS-A183S*-T_*ADH1*_	This study
pRS425-*AvGAS-A183T*	*LEU2*, pRS425-P_*GAL1*_- *AvGAS-A183T*-T_*ADH1*_	This study
pRS425-*AvGAS-A183G*	*LEU2*, pRS425-P_*GAL1*_- *AvGAS-A183G*-T_*ADH1*_	This study
pRS425-*AvGAS-A183L*	*LEU2*, pRS425-P_*GAL1*_- *AvGAS-A183L*-T_*ADH1*_	This study
pRS425-*AvGAS-L55F*	*LEU2*, pRS425-P_*GAL1*_- *AvGAS-L55F*-T_*ADH1*_	This study
RS425-*AvGAS- L55W*	*LEU2*, pRS425-P_*GAL1*_- *AvGAS-L55W*-T_*ADH1*_	This study
pRS425-*AvGAS- L55V*	*LEU2*, pRS425-P_*GAL1*_- *AvGAS-L55V*-T_*ADH1*_	This study
pRS425-*AvGAS- L55A*	*LEU2*, pRS425-P_*GAL1*_- *AvGAS-L55A*-T_*ADH1*_	This study
pRS425-*AvGAS- L55Y*	*LEU2*, pRS425-P_*GAL1*_- *AvGAS-L55Y*-T_*ADH1*_	This study
pRS425-*AvGAS- I75A*	*LEU2*, pRS425-P_*GAL1*_- *AvGAS-I75A*-T_*ADH1*_	This study
pRS425-*AvGAS- I75F*	*LEU2*, pRS425-P_*GAL1*_- *AvGAS-I75F*-T_*ADH1*_	This study
pRS425-*AvGAS- I75L*	*LEU2*, pRS425-P_*GAL1*_- *AvGAS-I75L*-T_*ADH1*_	This study
pRS425-*AvGAS- I75Y*	*LEU2*, pRS425-P_*GAL1*_- *AvGAS-I75Y*-T_*ADH1*_	This study
pRS425-*AvGAS-F23W*	*LEU2*, pRS425-P_*GAL1*_- *AvGAS-F23W*-T_*ADH1*_	This study
pRS425-*AvGAS-F23V*	*LEU2*, pRS425-P_*GAL1*_- *AvGAS-F23V*-T_*ADH1*_	This study
pRS425-*AvGAS-F23Y*	*LEU2*, pRS425-P_*GAL1*_- *AvGAS-F23Y*-T_*ADH1*_	This study
pRS425-*AvGAS-F23L*	*LEU2*, pRS425-P_*GAL1*_- *AvGAS-F23L*-T_*ADH1*_	This study
pRS425-*AvGAS-H152Y*	*LEU2*, pRS425-P_*GAL1*_- *AvGAS-H152Y*-T_*ADH1*_	This study
pRS425-*AvGAS-H152A*	*LEU2*, pRS425-P_*GAL1*_- *AvGAS-H152A*-T_*ADH1*_	This study
pRS425-*AvGAS-H152G*	*LEU2*, pRS425-P_*GAL1*_- *AvGAS-H152G*-T_*ADH1*_	This study
pRS425-*AvGAS-H152L*	*LEU2*, pRS425-P_*GAL1*_- *AvGAS-H152L*-T_*ADH1*_	This study
pRS425-*AvGAS-K218R*	*LEU2*, pRS425-P_*GAL1*_- *AvGAS-K218R*-T_*ADH1*_	This study
pRS425-*AvGAS-K218D*	*LEU2*, pRS425-P_*GAL1*_- *AvGAS-K218D*-T_*ADH1*_	This study
pRS425-*AvGAS-K218E*	*LEU2*, pRS425-P_*GAL1*_- *AvGAS-K218E*-T_*ADH1*_	This study
Strains		
*Saccharomyces cerevisiae* CEN.PK2-1D	*MATα*; *leu2-3*,*112*; *ura3-52; his3-*Δ*1*; *trp1-289*; *MAL2-8*^*C*^; *SUC2*	Laboratory stock
SGAS1	Expressing pRS425-*AmGAS* in CEN.PK2-1D	This study
SGAS2	Expressing pRS425-*ToGAS* in CEN.PK2-1D	This study
SGAS3	Expressing pRS425-*LsGAS* in CEN.PK2-1D	This study
SGAS4	Expressing pRS425-*NsGAS* in CEN.PK2-1D	This study
SGAS5	Expressing pRS425-*AvGAS* in CEN.PK2-1D	This study
SGAS6	Expressing pRS425-*NcGAS* in CEN.PK2-1D	This study
SGAS7	Expressing pRS425-*CsGAS* in CEN.PK2-1D	This study
SGAS8	Expressing pRS425-*MmGAS* in CEN.PK2-1D	This study
SGAS9	Expressing pRS425-*SpGAS* in CEN.PK2-1D	This study
SGAS10	Expressing pRS425-*PsGAS* in CEN.PK2-1D	This study
SMVA1	SGAS5; expressing pRS306*-tHMGR1*	This study
SMVA2	SMVA1; P_*ERG9*_Δ::*loxP-*P_*HXT1*_	This study
SMVA3	SMVA2; *DPP1*Δ::*loxP*; *LPP1*Δ::*loxP*	This study
SHEF	pRS306*-tHMGR1*; P_*ERG9*_::*loxP-*P_*HXT1*_; *DPP1*Δ::*loxP*; *LPP1*Δ::*loxP*	This study
SHEF-WT	Expressing pRS425-*AvGAS* in SHEF	This study
SHEF-L78A	Expressing pRS425-*AvGAS-L78A* in SHEF	This study
SHEF-L78F	Expressing pRS425-*AvGAS-L78F* in SHEF	This study
SHEF-L78T	Expressing pRS425-*AvGAS-L78T* in SHEF	This study
SHEF-L78I	Expressing pRS425-*AvGAS-L78I* in SHEF	This study
SHEF-L78G	Expressing pRS425-*AvGAS-L78G* in SHEF	This study
SHEF-L78V	Expressing pRS425-*AvGAS-L78V* in SHEF	This study
SHEF-A181C	Expressing pRS425-*AvGAS-A181C* in SHEF	This study
SHEF-A181G	Expressing pRS425-*AvGAS-A181G* in SHEF	This study
SHEF-A181F	Expressing pRS425-*AvGAS-A181F* in SHEF	This study
SHEF-A181L	Expressing pRS425-*AvGAS-A181L* in SHEF	This study
SHEF-A181Y	Expressing pRS425-*AvGAS-A181Y* in SHEF	This study
SHEF-A181T	Expressing pRS425-*AvGAS-A181Y* in SHEF	This study
SHEF-A182T	Expressing pRS425-*AvGAS-A182T* in SHEF	This study
SHEF-A182G	Expressing pRS425-*AvGAS-A182G* in SHEF	This study
SHEF-A182S	Expressing pRS425-*AvGAS-A182S* in SHEF	This study
SHEF-A182F	Expressing pRS425-*AvGAS-A182F* in SHEF	This study
SHEF-A182L	Expressing pRS425-*AvGAS-A182L* in SHEF	This study
SHEF-A183F	Expressing pRS425-*AvGAS-A183F* in SHEF	This study
SHEF-A183S	Expressing pRS425-*AvGAS-A183S* in SHEF	This study
SHEF-A183T	Expressing pRS425-*AvGAS-A183T* in SHEF	This study
SHEF-A183G	Expressing pRS425-*AvGAS-A183G* in SHEF	This study
SHEF-A183L	Expressing pRS425-*AvGAS-A183G* in SHEF	This study
SHEF-L55F	Expressing pRS425-*AvGAS-L55F* in SHEF	This study
SHEF-L55W	Expressing pRS425-*AvGAS- L55W* in SHEF	This study
SHEF-L55V	Expressing pRS425-*AvGAS-L55V* in SHEF	This study
SHEF-L55A	Expressing pRS425-*AvGAS-L55A* in SHEF	This study
SHEF-L55Y	Expressing pRS425-*AvGAS-L55Y* in SHEF	This study
SHEF- I75A	Expressing pRS425-*AvGAS-I75A* in SHEF	This study
SHEF-I75F	Expressing pRS425-*AvGAS-I75F* in SHEF	This study
SHEF-I75L	Expressing pRS425-*AvGAS-I75L* in SHEF	This study
SHEF-I75Y	Expressing pRS425-*AvGAS-I75Y* in SHEF	This study
SHEF-F23W	Expressing pRS425-*AvGAS-F23W* in SHEF	This study
SHEF-F23V	Expressing pRS425-*AvGAS-F23V* in SHEF	This study
SHEF-F23Y	Expressing pRS425-*AvGAS-F23Y* in SHEF	This study
SHEF-F23L	Expressing pRS425-*AvGAS-F23L* in SHEF	This study
SHEF-H152Y	Expressing pRS425-*AvGAS-H152Y* in SHEF	This study
SHEF-H152A	Expressing pRS425-*AvGAS-H152A* in SHEF	This study
SHEF-H152G	Expressing pRS425-*AvGAS-H152G* in SHEF	This study
SHEF-H152L	Expressing pRS425-*AvGAS-H152L* in SHEF	This study
SHEF-K218R	Expressing pRS425-*AvGAS-K218R* in SHEF	This study
SHEF-K218D	Expressing pRS425-*AvGAS-K218D* in SHEF	This study
SHEF-K218E	Expressing pRS425-*AvGAS-K218E* in SHEF	This study

### Homology modelling

A high resolution protein structure with significant homology to the *Av*GAS protein sequence was obtained from the Protein Data Bank (PDB) using NCBI BLAST. This structure (PDB ID: 5NX6) was used as a template for subsequent homology modelling which was performed with the Molecular Operating Environment (MOE) 2014.09 software package. After removing uncorrelated ligands, target and template sequences were compared to define conserved regions. Ten transition models were obtained by permutation and combination of candidate loop regions as well as side-chain rotamers. These ten candidates were then subjected to energy optimization with the AMBER12/EHT force field and R-field implicit solvent. The quality of each candidate was evaluated using the GB/VI scale. The highest-scoring model was chosenfor further energy optimized.

### Molecular docking

The docking module Dock in the MOE software was used to predict the binding between *Av*GAS and the substrate FPP. The 2D structure of FPP was drawn with ChemBioDraw 2014, and a 3D structure was obtained with MOE by energy optimization. The protonation state of proteins and the location of hydrogen atoms was obtained using the LigX module in MOE, at pH 7 and 300 K. Before molecular docking, the AMBER12:EHT force field and the R-field implicit solvent model were selected. Docking was completed in the induced fit mode; the side chains in the binding pocket were automatically adjusted according to ligand conformation. The weight that restrained the rotation of the side chains was set at 10. The binding modes of FPP were first ranked by a London dG scoring function. The first 30 conformations were re-assessed following the GBVI/WSA dG method. The interactions between FPP and proteins were graphically represented using PyMOL (www.pymol.org).

### Analysis of germacrene A production by engineered yeast strains

Recombinant yeast transformants were inoculated into 5 mL of SC liquid media containing 1% glucose as carbon source. After 24 h growth, equal amount of yeast cells were collected and transferred to 30 mL PY (2% peptone plus 1% yeast extract) liquid media containing 0.1% glucose and 2% galactose, with initial OD_600_ of 0.5 measured by a microplate reader (BioTek, USA). All flasks were immediately supplemented with 20% (vol/vol) dodecane after seeding. After 72-h cultivation, 100 μL of cell culture was taken and diluted for measuring OD_600_ using the microplate reader (BioTek, USA). The rest was centrifuged at 13,000 rpm for 20 min, and 0.5 mL of the upper dodecane layer were collected and stored at -20 °C. To analyze whether germacrene A was trapped intracellularly, 30 mL of SHEF yeast cells were collected, washed with 25 mM phosphate buffer at pH 7.5, and resuspended in the same buffer containing 25 U lyticase. After treatment at 30 °C for 30 min, cells were collected and resuspended, followed by disruption using glass beads in a bead beater with 6000 rpm for 15 s, for three times. Cell lysates were subject to centrifugation at 8000 rpm for 10 min, and the supernatant was separated, with an addition of 50% dodecane (vol/vol). The mixture was vortexed for 10 min, and then centrifuged at 13,000 rpm for 20 min. The final upper dodecane layer were collected and stored. ß-elemene were identified using a GC–MS system (Shimadzu Co., Kyoto, Japan) equipped with an RTX-1 column (30 m × 0.25 mm × 0.25 μm). One microliter of each dodecane sample was injected into the system with a split ratio of 10 and the carrier gas helium was set at a constant flow rate of 0.78 mL/min. The oven temperature was first maintained at 40 °C for 2 min, and then gradually increased to 160 °C at a rate of 10 °C /min, held for 2 min, and finally increased to 250 °C at a rate of 15C /min and held for 5 min. The mass spectrometer was set to the SIM acquisition mode, scanning m/z ions within the range 50–650 for identification of ß-elemene. For quantification of ß-elemene, a GC-FID system (Shimadzu Co., Kyoto, Japan) equipped with an RTX-1701 column (30 m × 0.25 mm × 0.25 μm) was applied. One microliter of each dodecane sample was injected into the system with a split ratio of 10 and the carrier gas nitrogen was set at a constant flow rate of 2.34 mL/min. The oven temperature was first maintained at 40 °C for 1 min, and then gradually increased to 180 °C at a rate of 25 °C /min, held for 3 min, and finally increased to 250 °C at a rate of 15 °C/min and held for 5 min. The total run time was 20 min. ß-elemene standard was dissolved in dodecane and used to plot standard curves for quantification. Germacrene A production are presented as ß-elemene equivalents.

### Sequence analysis and phylogenetic tree construction

The amino acid sequences of GASs were retrieved from NCBI or Uniprot databases. Sequence alignments were performed using Clustal W [34]. The phylogenetic tree was constructed using the neighbour-joining method with MEGA7.0 [35]. Numbers on the tree branches represent the bootstrap support calculated per 1000 bootstrap replicates.

## Conclusions

By combining GAS screening, enzyme mutagenesis engineering, and metabolic engineering, production of germacrene A in yeast was markedly improved. GAS enzyme screening showed that three cyanobacterial GASs, especially *Av*GAS, outperformed plant orthologues in germacrene A production in yeast. Simultaneously metabolic engineering of yeast strain to enhance the MVA pathway flux and FPP supply dramatically improved germacrene A production by 11-fold. Finally, site-directed mutation of *Av*GAS Phe23 to tryptophan or valine led to a further 35.2% and 21.8% increase in germacrene A accumulation. These results highlight the potential of bacterial terpene synthases in sesquiterpene production in engineered yeast and provide insights into the mutagenesis engineering of sesquiterpene synthases.

## Supplementary Information

**Additional file 1:** **Figure S1.** Identification of germacrene A by GC-MS analysis. (A, B) GC analyses of the ß-elemene standard (A) and the culture extract of the yeast strain SGAS5. (C) GC-MS spectra of the chromatographic peak corresponding to ß-elemene in (B). **Figure S2.** List of pairwise protein sequence identity (%) comparison between ten GAS candidates applied for screening in this study. **Figure S3.** Extracellular farnesol production of yeast strains SGAS5, SMVA1, SMVA2, and SMVA3. **Figure S4.** Sequence alignment of *Av*GAS with *Ns*GAS, *Nc*GAS, and bLinS from *Streptomyces clavuligerus* (5NX6). Two conserved regions within the active site, the aspartate-rich (DDXX(X)(D,E)) motif and the NSE (NDXXSXX(R,K)(E,D)) triad, required for binding catalytically essential Mg^2+^ ions, were indicated with boxes. **Figure S5.** Extracellular germacrene A production of yeast cells expressing the indicated GASs, respectively. GASs were expressed in *S. cerevisiae* CEN.PK2-1D wherein *tHMGR1* was overexpressed and *ERG9* was downregulated via promoter replacement, as described in the main text. **Figure S6.** Extracellular farnesol production of yeast strain SHEF and SHEF strains expressing *Ls*GAS, *Av*GAS, and *Av*GAS-F23W, respectively. **Figure S7.** Extracellular and intracellular germacrene A accumulation in the yeast strain SHEF after cultivation for 72 h. **Figure S8.** Location of Phe23 in the modelled structure of *Av*GAS. The substrate FPP is represented as cyan sticks and Phe23 is shown as purple sticks. **Table S1.** Growth analyses of yeast cells expressing Phe23 mutants of *Av*GAS after cultivation for 72 h.

## Data Availability

All data generated or analyzed during this study are included in this published article.

## Abbreviations

GAS: Germacrene A synthase
MVA: Mevalonate
tHMGR1: Truncated HMG-CoA reductase 1
FPP: Farnesyl pyrophosphate
LB: Luria–Bertani
YPD: Yeast extract peptone dextrose
SC: Synthetic complete
PDB: Protein Data Bank
MOE: Molecular Operating Environment
GC: Gas chromatography
MS: Mass spectroscopy
