# Supplementary material for: Improved production of germacrene A, a direct precursor of ß-elemene, in engineered Saccharomyces cerevisiae by expressing a cyanobacterial germacrene A synthase
Source: Microb Cell Fact. 2021 Jan 7;20:7. doi: 10.1186/s12934-020-01500-3 (PMC7791714; doi:10.1186/s12934-020-01500-3)
Supplement: Supplementary file 1 — Additional file 1: Figure S1. Identification of germacrene A by GC-MS analysis. (A, B) GC analyses of the ß-elemene standard (A) and the culture extract of the yeast strain SGAS5. (C) GC-MS spectra of the chromatographic peak corresponding to ß-elemene in (B). Figure S2. List of pairwise protein sequence identity (%) comparison between ten GAS candidates applied for screening in this study. Figure S3. Extracellular farnesol production of yeast strains SGAS5, SMVA1, SMVA2, and SMVA3. Figure S4. Sequence alignment of AvGAS with NsGAS, NcGAS, and bLinS from Streptomyces clavuligerus (5NX6). Two conserved regions within the active site, the aspartate-rich (DDXX(X)(D,E)) motif and the NSE (NDXXSXX(R,K)(E,D)) triad, required for binding catalytically essential Mg2+ ions, were indicated with boxes. Figure S5. Extracellular germacrene A production of yeast cells expressing the indicated GASs, respectively. GASs were expressed in S. cerevisiae CEN.PK2-1D wherein tHMGR1 was overexpressed and ERG9 was downregulated via promoter replacement, as described in the main text. Figure S6. Extracellular farnesol production of yeast strain SHEF and SHEF strains expressing LsGAS, AvGAS, and AvGAS-F23W, respectively. Figure S7. Extracellular and intracellular germacrene A accumulation in the yeast strain SHEF after cultivation for 72 h. Figure S8. Location of Phe23 in the modelled structure of AvGAS. The substrate FPP is represented as cyan sticks and Phe23 is shown as purple sticks. Table S1. Growth analyses of yeast cells expressing Phe23 mutants of AvGAS after cultivation for 72 h. [file 12934_2020_1500_MOESM1_ESM.docx]

**Improved production of germacrene A, a direct precursor of ß-elemene, in engineered *Saccharomyces cerevisiae* by expressing a cyanobacterial germacrene A synthase**

Weixin Zhang^1^, Junqi Guo^1^, Zheng Wang^1^, Yanwei Li^2^, Xiangfeng Meng^1^, Yu Shen^1^, Weifeng Liu^1^*

^1^State Key Laboratory of Microbial Technology, Shandong University, No.72 Binhai Road, Qingdao 266237, P. R. China

^2^Environment Research Institute, Shandong University, Qingdao, 266237, P. R. China.

^*^Correspondence should be addressed to W Liu.

E-mail: weifliu@sdu.edu.cn

**Additional Data**


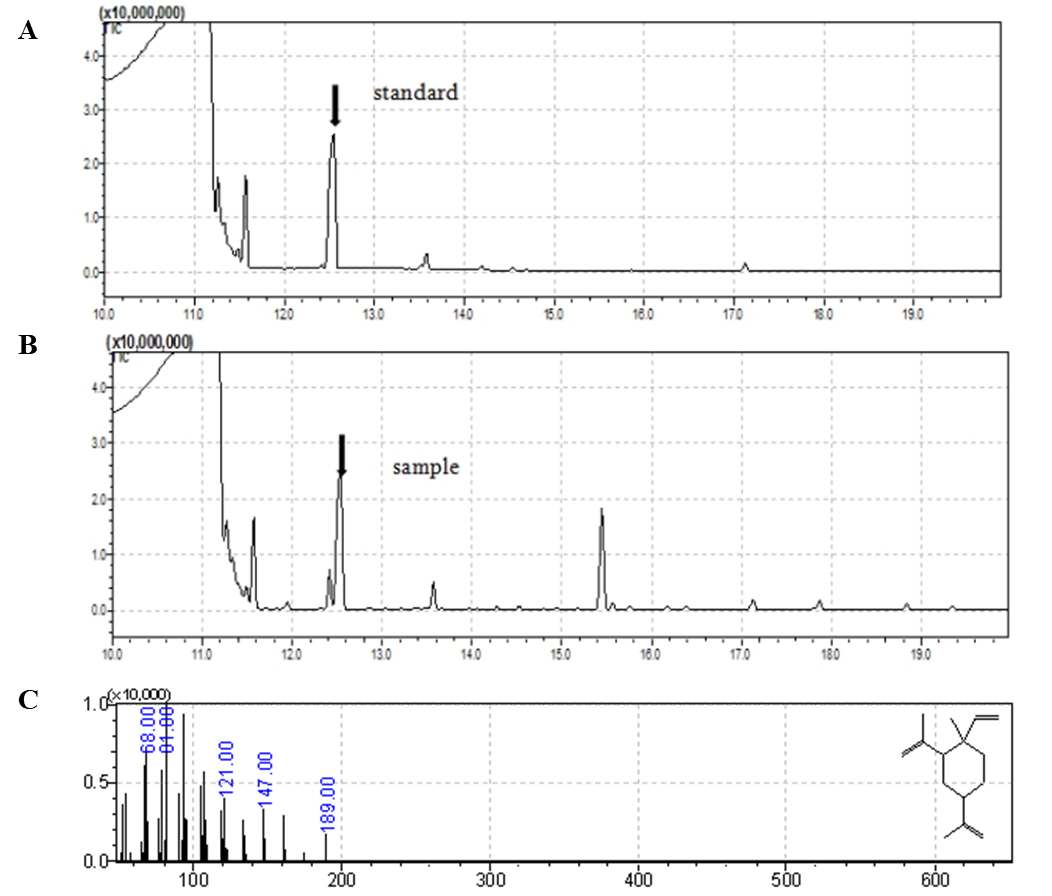


Figure S1 Identification of germacrene A by GC-MS analysis. (A-B) GC analyses of the ß-elemene standard (A) and the culture extract of the yeast strain SGAS5. (C) GC-MS spectra of the chromatographic peak corresponding to ß-elemene in (B).


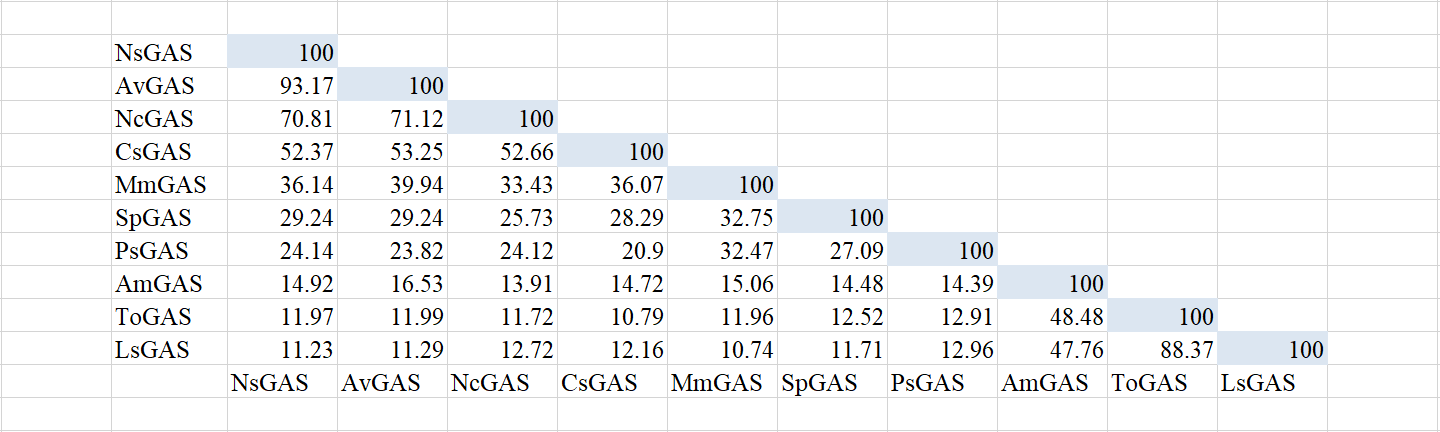


Figure S2 List of pairwise protein sequence identity (%) comparison between ten GAS candidates applied for screening in this study.


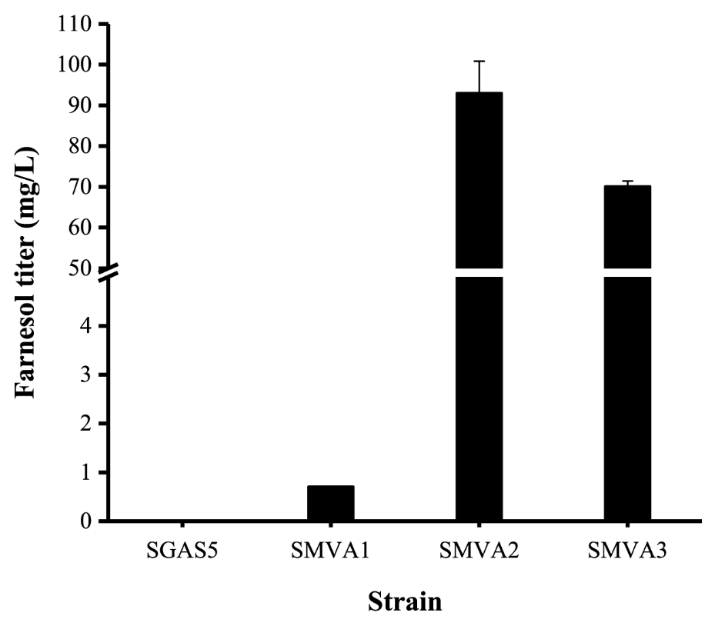


Figure S3 Extracellular farnesol production of yeast strains SGAS5, SMVA1, SMVA2, and SMVA3.


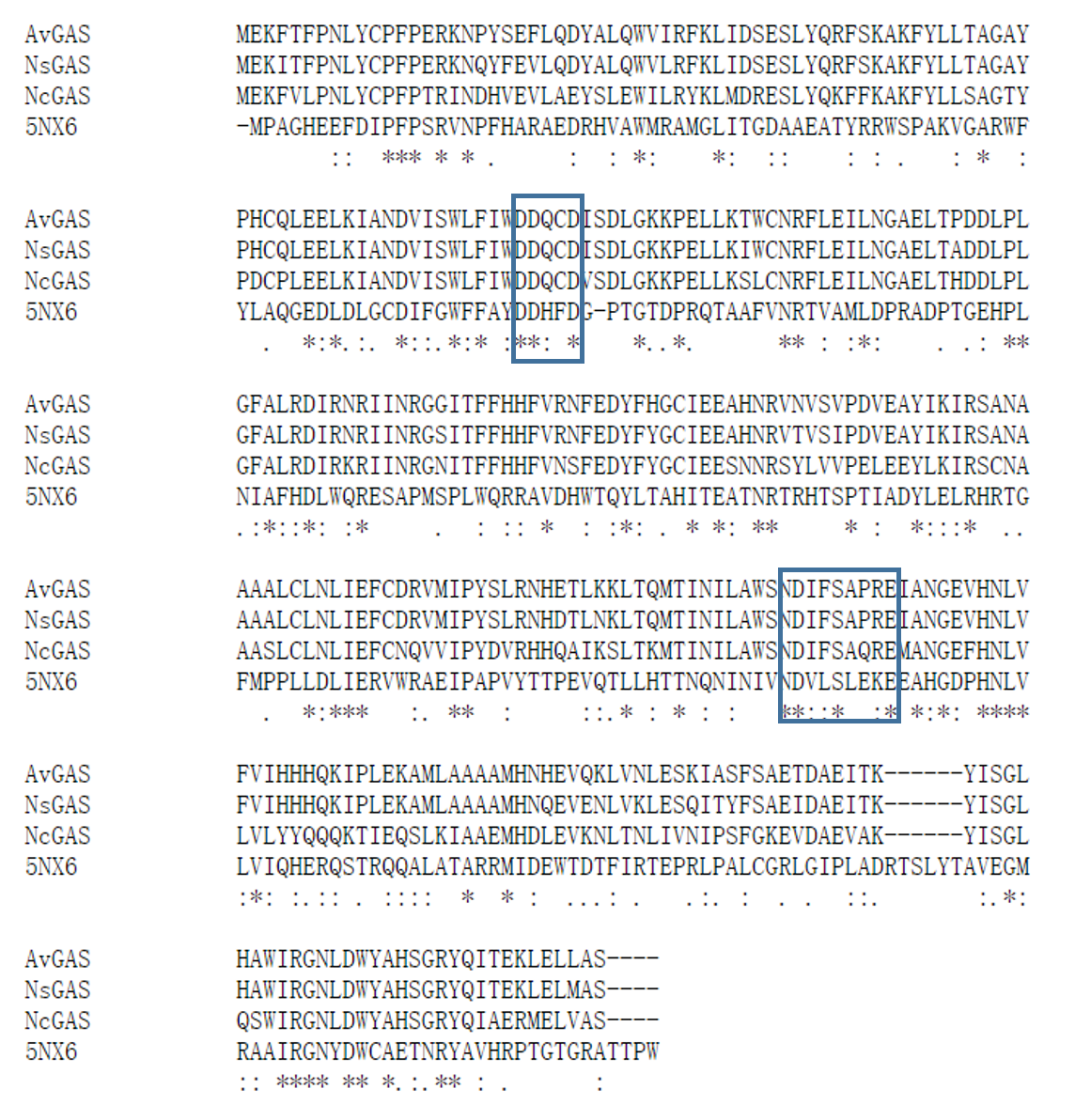


Figure S4 Sequence alignment of *Av*GAS with *Ns*GAS, *Nc*GAS, and bLinS from *Streptomyces clavuligerus* (5NX6). Two conserved regions within the active site, the aspartate-rich (DDXX(X)(D,E)) motif and the NSE (NDXXSXX(R,K)(E,D)) triad, required for binding catalytically essential Mg^2+^ ions, were indicated with boxes.





Figure S5 Extracellular germacrene A production of yeast cells expressing the indicated GASs, respectively. GASs were expressed in *S. cerevisiae* CEN.PK2-1D wherein *tHMGR1* was overexpressed and *ERG9* was downregulated via promoter replacement, as described in the main text.


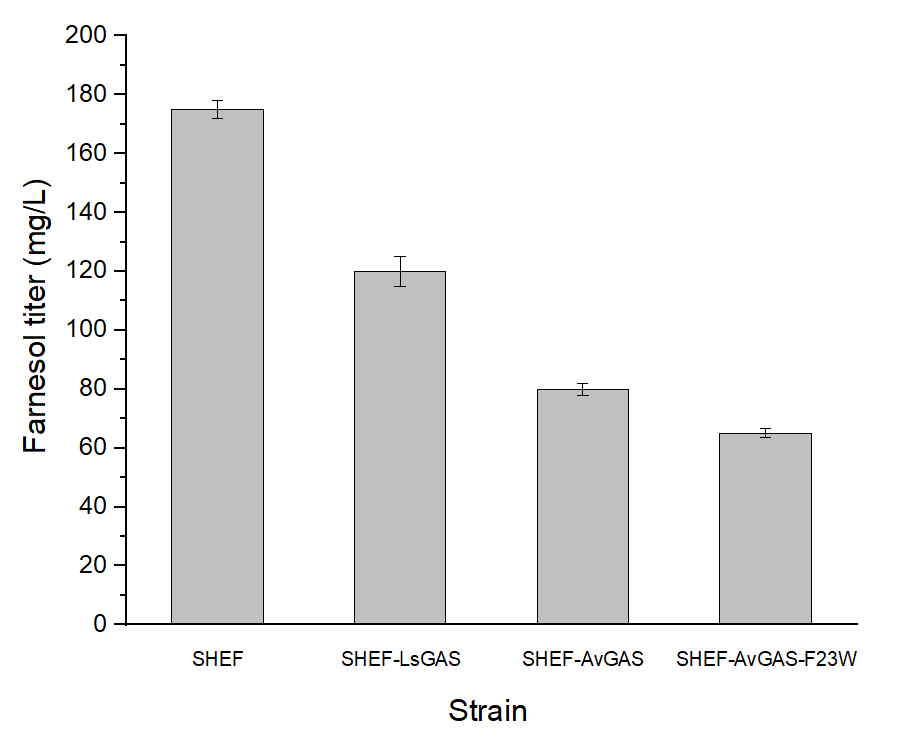


Figure S6 Extracellular farnesol production of yeast strain SHEF and SHEF strains expressing *Ls*GAS, *Av*GAS, and *Av*GAS-F23W, respectively.


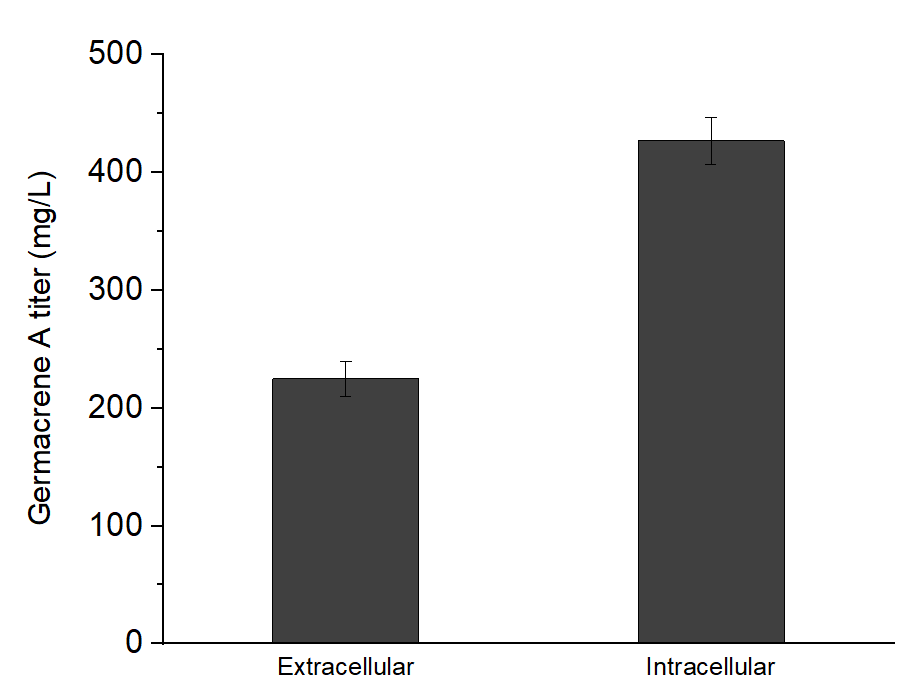


Figure S7 Extracellular and intracellular germacrene A accumulation in the yeast strain SHEF after cultivation for 72 h.


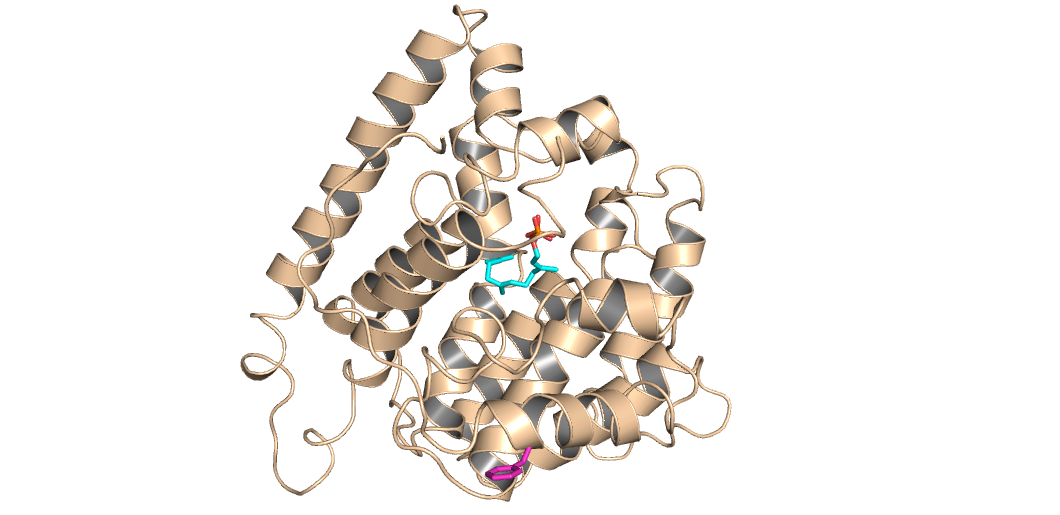


Figure S8 Location of Phe23 in the modelled structure of *Av*GAS. The substrate FPP is represented as cyan sticks and Phe23 is shown as purple sticks.

Table S1 Growth analyses of yeast cells expressing Phe23 mutants of *Av*GAS after cultivation for 72 h.

| Mutants | OD_600_^a^ |
| --- | --- |
| SHEF-WT | 5.501±0.02 |
| SHEF-F23W | 5.256±0.02 |
| SHEF-F23V | 5.311±0.02 |
| SHEF-F23Y | 5.667±0.02 |
| SHEF-F23L | 5.593±0.02 |

^a^ OD600 was measured using a microplate reader (BioTek, USA).
